# From pathogen to prognosis: microbial keratitis spectrum and treatment outcomes in a 5-Year tertiary center experience

**DOI:** 10.1186/s12348-025-00524-3

**Published:** 2025-12-30

**Authors:** Bagim Aycin Cakir Ince, Onder Ayyildiz, Gokhan Ozge

**Affiliations:** 1 Gulhane Training and Research Hospital, Etlik, Gen. Dr. Tevfik Sağlam St. No:1, 06010, Ankara, Türkiye; 2 Dünyagöz Hospital, İzmir, Türkiye

**Keywords:** Empirical treatment, Endophthalmitis, Microbial keratitis, Penetrating keratoplasty, Topical antibiotics, Vitrectomy

## Abstract

**Background:**

Microbial keratitis is a vision-threatening corneal infection frequently encountered in tertiary eye care settings. Early identification of risk factors and prompt empirical treatment are essential for preventing permanent visual impairment and maintaining anatomical integrity. This study aimed to define the clinical and microbiological profiles of microbial keratitis and to evaluate empirical treatment responses and surgical outcomes.

**Methods:**

This was a descriptive, retrospective study conducted through a review of clinical records over a five-year period (2020–2025). A total of 73 patients diagnosed with microbial keratitis at a tertiary eye care center were included. Data on demographic characteristics, predisposing risk factors, clinical presentation, microbiological culture results, and treatment modalities were collected. The patients’ responses to empirical medical therapy and the need for surgical interventions, including penetrating keratoplasty, pars plana vitrectomy and evisceration were evaluated.

**Results:**

Positive microbial cultures were obtained in 58.9% of the cases. Among these, *Staphylococcus spp.* was the most commonly isolated organism (36.9%), followed by *Streptococcus spp.* (21.7%), *Klebsiella spp.* (11.0%), *Pseudomonas aeruginosa* (9.0%) and fungal pathogens (4.3%). Trauma was identified as the most frequent predisposing risk factor across all age groups. Clinical improvement was observed in 65.8% of patients following topical empirical therapy. Surgical intervention was required in 25 patients due to clinical deterioration, including penetrating keratoplasty, re-keratoplasty, pars plana vitrectomy and evisceration. Endophthalmitis developed in 7 patients (9.5%) with causative organisms including *Staphylococcus* (44.4%), *Streptococcus* (33.3%), *Klebsiella* (11.1%) and *Pseudomonas aeruginosa* (11.1%). The anatomical success rate following treatment was 85.7%.

**Conclusions:**

Initiation of empirical therapy targeting regionally prevalent pathogens may may contribute to better visual outcomes in selected cases. In cases unresponsive to medical management, early intervention with penetrating keratoplasty prior to limbal involvement or corneal perforation may improve prognosis and reduce the incidence of severe complications such as endophthalmitis.

## Introduction

Infectious keratitis is a potentially serious ophthalmic condition that may lead to visual impairment if not managed appropriately. It frequently limits visual rehabilitation, especially in advanced cases. The clinical diagnosis is based on the presence of corneal epithelial defect with underlying stromal infiltration, often accompanied by conjunctival hyperemia, anterior chamber reaction, and, in some cases, hypopyon. Infectious keratitis can be broadly classified into microbial keratitis caused by bacteria, fungi, or protozoa and viral keratitis [1]. Infectious keratitis most commonly occurs in the presence of conditions that disrupt the cornea’s natural defense mechanisms. Such conditions include ocular surface diseases, contact lens use, and ocular trauma. These factors compromise corneal integrity and facilitate microbial invasion. A major challenge in managing microbial keratitis is the difficulty in early differentiation between bacterial, fungal, and polymicrobial infections, which may delay appropriate therapy [2].

Epidemiological studies play a key role in identifying microorganism-specific risk factors and clinical presentations, thereby guiding diagnosis and treatment strategies for infectious keratitis. However, obtaining reliable data remains challenging, as infectious keratitis is often grouped under the broader classification of ‘corneal blindness’, which also encompasses traumatic, inflammatory, and hereditary corneal conditions [1].

To address this gap, the present retrospective study was conducted to provide updated epidemiological and clinical insights by analyzing the demographic characteristics, risk factors, and microbiological profiles of patients diagnosed with microbial keratitis over a five-year period. The study also evaluated initial empirical treatment approaches and their clinical outcomes.

## Methods

Medical records of patients diagnosed with keratitis and who underwent microbiological culture testing at our tertiary eye care center between 2020 and 2025 were retrospectively reviewed. The study adhered to the tenets of the Declaration of Helsinki and was approved by the institutional ethics committee. Informed consent for the use of anonymized medical data was obtained from all patients.

The following clinical and demographic data were collected: age, gender, duration of follow-up, visual acuity at the time of keratitis diagnosis and after treatment, history of risk factors, types of microorganisms identified through culture, and the medical treatments administered. In addition, documentation included the presence of corneal ulceration, perforation, or abscess; indications for surgical intervention; the specific surgical techniques performed; postoperative complications; and rates of re-keratoplasty (rPKP).

Preoperative and postoperative visual acuity was evaluated using standard measures, including light perception (LP), hand motion (HM), finger counting (CF), and best-corrected distance visual acuity (BCVA) assessed by the Snellen chart. For statistical analysis, low vision states were converted into LogMAR equivalents using the following values: CF = 1.9, HM = 2.3, LP = 2.7, and no light perception (NLP) = 3.0 [3].

This study included only patients with microbial keratitis who had not received prior antibiotic treatment and from whom microbiological culture samples were obtained. Corneal cultures were not performed, and such patients were not included in the study, if they had already started antibiotic treatment at external centers and showed clinical improvement. On the other hand, seven patients who were prescribed topical corticosteroids without microbiological confirmation were included in the study. All patient data were obtained from the institutional Electronic Health Record (EHR) system, where microbial keratitis cases are registered with a dedicated diagnostic code. This allowed accurate identification of cases and correlation with treatment outcomes.

As stated in previous reference works [4, 5], corneal cultures were obtained in the following clinical situations:

I. The corneal infiltrate is centrally located, larger than 2 mm, and/or associated with significant stromal involvement or melting.
II. The infection is chronic or does not respond to broad-spectrum antibiotic therapy.
III. There is a documented history of previous corneal surgeries.
IV. Atypical clinical features suggest the possibility of fungal, amoebic, or mycobacterial keratitis.
V. Infiltrates appear in multiple locations on the cornea.

For microbiological diagnosis, corneal swabs were obtained directly from the site of active infection. Samples were promptly sent to the microbiology laboratory for direct microscopy, culture, and antimicrobial susceptibility testing. In cases where a viral etiology was suspected, corneal specimens were additionally submitted for polymerase chain reaction (PCR) analysis.

Severe bacterial keratitis was defined by central infiltrates larger than 2 mm, deep stromal involvement, presence of hypopyon, stromal thinning, or rapid clinical progression. Mild keratitis was characterized by small peripheral infiltrates, minimal stromal involvement, and absence of anterior chamber reaction.

Patients presenting with severe keratitis were empirically treated with fortified topical vancomycin (50 mg/mL) and ceftazidime (50 mg/mL), combined with bacitracin-neomycin ophthalmic ointment. Patients with milder bacterial keratitis received topical moxifloxacin hydrochloride (a fourth-generation fluoroquinolone). In both treatment groups, the same bacitracin-neomycin ointment was additionally prescribed as part of the standard regimen.

In two cases of keratitis secondary to herpes simplex virus, topical ganciclovir gel (1.5 mg/g) was administered five times daily, and oral valacyclovir (1000 mg) was given three times daily. These treatments were used in combination with topical moxifloxacin.

In cases with clinical signs suggestive of fungal keratitis such as feathery-edged infiltrates, satellite lesions, immune ring formation, delayed epithelial healing and a history of trauma with organic material (e.g., tree branch), empirical topical antifungal therapy was initiated prior to microbiological confirmation. For cases of fungal keratitis, topical fortified amphotericin B (0.5 mg/mL, hourly) was combined with oral voriconazole (200 mg, twice daily).

Empirical antibiotic treatment was initiated with one drop every 30 min during the first two hours, followed by hourly administration on the first day. Adjustments to treatment regimens were made based on microbiological findings and antimicrobial susceptibility results.

Clinical improvement was defined as a reduction in the size of the corneal infiltration, decreased lesion depth with more clearly demarcated borders, resolution or reduction of anterior chamber reaction (if present), and improvement in visual acuity.

For surgically managed patients, complete eradication of the primary infection and preservation of globe integrity were considered indicators of treatment success.

The development of endophthalmitis was classified as treatment failure. In cases unresponsive to intravitreal vancomycin and ceftazidime, pars plana vitrectomy (PPV) was performed.

Following penetrating keratoplasty (PKP), topical antimicrobial therapy was continued for a minimum of 4 to 8 weeks, guided by antibiogram sensitivity results. Topical 1% prednisolone was initiated twice daily, gradually increased to 4–6 times daily, and then tapered over a period of 3 to 6 months based on clinical progress. In addition, artificial tears were prescribed to all patients, with instillation every two hours.

For patients who underwent PPV notable, a similar topical antimicrobial regimen was continued for a minimum of 4 to 8 weeks, based on individual antibiogram sensitivity results. Topical 1% prednisolone was initially administered twice daily, increased to four times daily, and subsequently tapered over a period of up to two months, depending on clinical response. Additionally, anti-glaucoma topical therapy was prescribed to six patients, none of whom required surgical intervention for intraocular pressure control.

### Statistical analysis

Statistical analyses were performed using SPSS software (IBM Corp., Armonk, NY, USA). Continuous variables were expressed as mean ± standard deviation (SD), and categorical variables were presented as percentages. The chi-square test was used to evaluate associations between categorical variables, and the independent samples t-test was applied for comparisons of continuous variables. A p-value of < 0.05 was considered statistically significant.

## Results

**Fig. 1:**
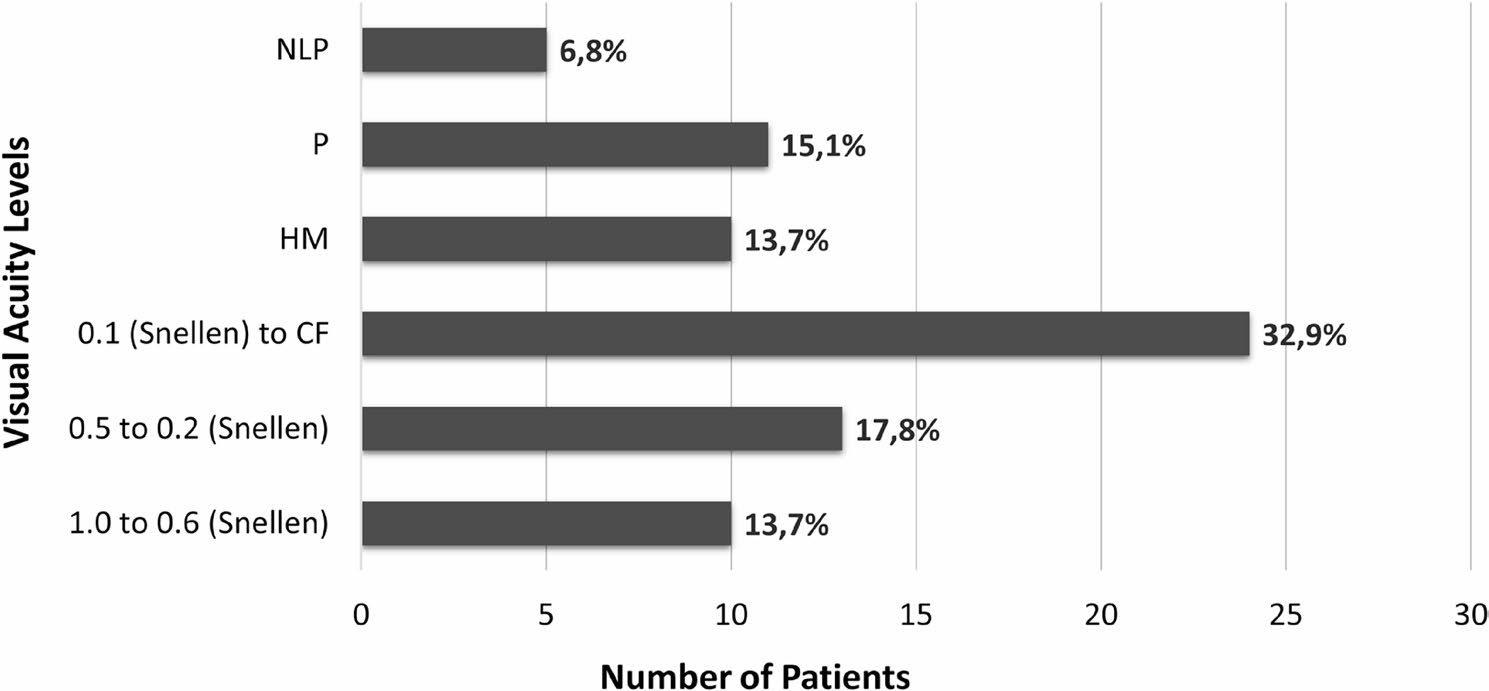
Baseline visual acutiy of patients with keratitis prior to culture collection

The most frequently reported presenting symptoms were ocular redness, pain, epiphora, and blurred vision. In patients diagnosed with keratitis, baseline visual acuity assessed prior to culture collection is shown in Fig. 1.

Based on BCVA comparison before and after treatment, mean LogMAR values improved from 1.6 to 1.2, representing a statistically significant gain in visual acuity (*p* = 0.034).

**Fig. 2:**
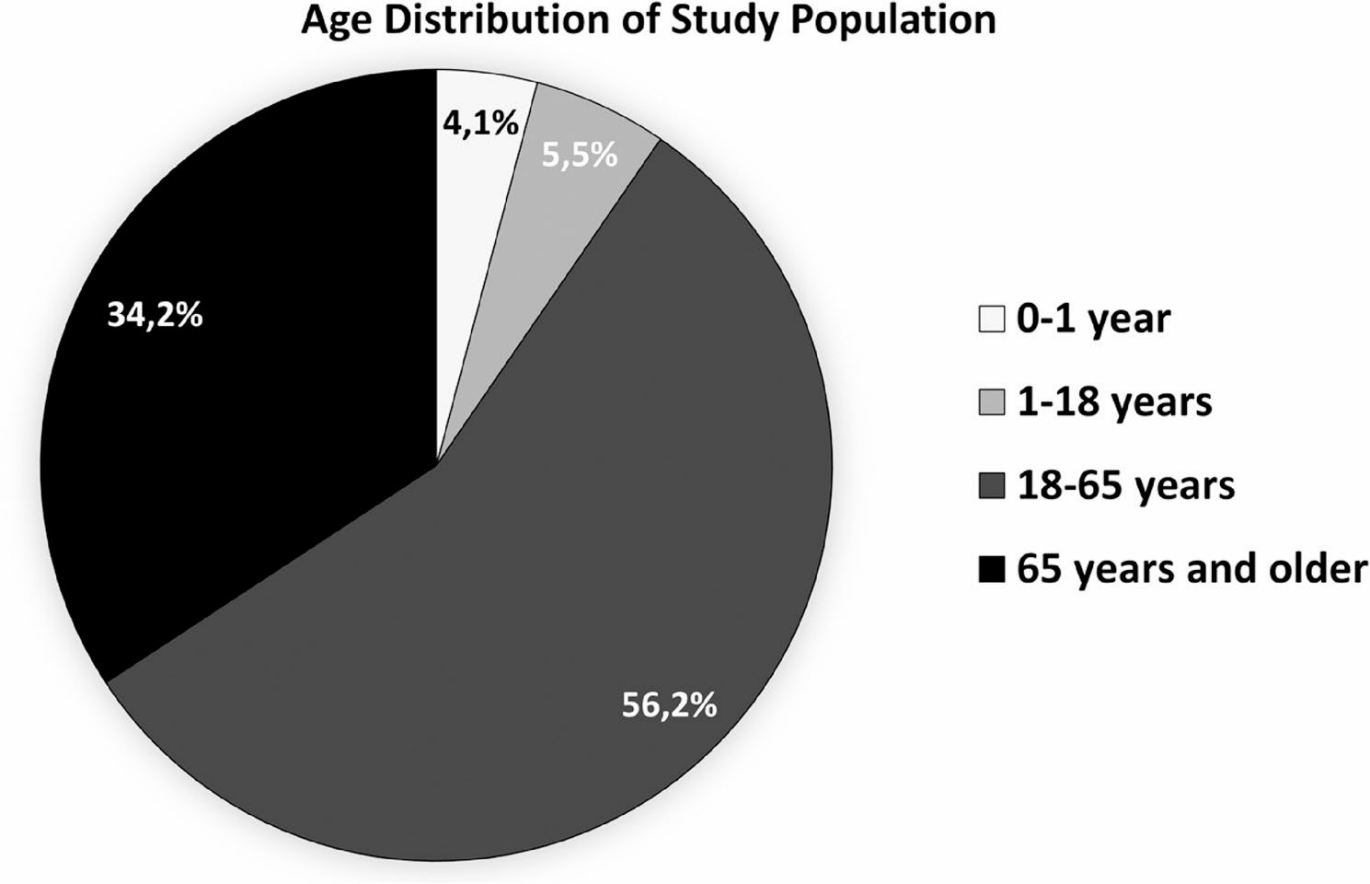
Age distribution of patients included in the study

Of the 73 patients included in the study, 69.9% were male and 30.1% were female. The mean age of the study population was 48.95 ± 24.24 years. The age distribution of the patients is illustrated in Fig. 2.

Based on patient history, the most frequently identified risk factor was ocular trauma, which included foreign body injury, chemical exposure and sequelae of penetrating injury or previous ocular surgery. Other notable risk factors included contact lens use, prior herpes simplex virus infection and prolonged hospitalization in an intensive care unit, often associated with immunosuppression or exposure keratopathy. Review of medical records revealed that 18 patients (24.7%) had a documented history of diabetes mellitus, a systemic condition known to impair corneal healing.

Additionally, 12 patients had received initial treatment at other healthcare facilities before presenting to our hospital. Among these, 7 patients were prescribed topical corticosteroids by primary care providers without microbiological confirmation, which may have contributed to disease progression and clinical deterioration. This highlights the importance of microbiological diagnosis prior to initiating corticosteroid therapy.

Cultural growth was observed in 47 out of 73 patients (64.38%). Among these culture-positive cases, 42 (89.36%) demonstrated monomicrobial growth, while 5 cases (10.64%) were identified as polymicrobial, as shown in Table 1.

**Table 1:** Demographic parameters of infectious keratitis categorized by microbial profile

Variable	No growth (N=26)	Monomicrobial (N=42)	Polymicrobial (N=5)
Age (Mean ± SD)	54 ± 21	43 ± 26	62 ± 11
Gender			
Male	18(%24.65)	28(%38,35)	3(%4,11)
Female	8(%10,95)	14(%19,2)	2(%2.74)

The identified organisms were categorized as follows: approximately 61.7% were Gram-positive bacteria, 34.0% were Gram-negative bacteria, and 4.3% were fungal pathogens.

The most frequently isolated Gram-positive organism was *Staphylococcus spp.* (36.2%), with *Staphylococcus epidermidis* accounting for 12.76% of these cases. *Streptococcus spp.* was the second most common Gram-positive group, representing 21.3% of isolates. Within this group, *Streptococcus pneumoniae* and *Streptococcus pyogenes* were the most frequently identified species, each comprising 4.25%. Among Gram-negative pathogens, *Klebsiella spp.* was the most isolated genus (10.64%) with *Klebsiella oxytoca* being the predominant species. *Pseudomonas aeruginosa* was the second most frequent Gram-negative isolate, observed in 8.51% of cases. In the pediatric subgroup, two infants admitted to the intensive care unit were found to have cultures positive for *Pseudomonas aeruginosa*. One infant responded to medical treatment, while the other developed endophthalmitis and subsequently required PPV. Regarding fungal pathogens, one isolate each of *Candida* and *Fusarium* species was identified **(**Tables 2, 3 and 4**).**

**Table 2:** Causative agents of infectious keratitis categorized by microbial profile: Gram-Positive Organisms

	**Number of Patients**	**Microorganisms/Overall**
Gram-Positive Organisms	**29**	**61,70%**
Staphylococcus	**17**	**36,17%**
Staphylococcus epidermidis	6	12,77%
Staphylococcus aureus	5	10,64%
Staphylococcus hominis	2	4,26%
Staphylococcus capitis	2	4,26%
Staphylococcus oralis	1	2,13%
Staphylococcus pasteuri	1	2,13%
Streptococcus	**10**	**21,28%**
Streptococcus pneumoniae	2	4,26%
Streptococcus pyogenes	2	4,26%
Streptococcus mitis	1	2,13%
Streptococcus oralis	1	2,13%
Streptococcus anginosus	1	2,13%
Streptococcus intermedius	1	2,13%
Streptococcus parasanguinis	1	2,13%
Streptococcus spescies	1	2,13%
Propionibacterium acnes	**1**	**2,13%**
Enterococcus faecalis	**1**	**2,13%**

**Table 3:** Causative agents of infectious keratitis categorized by microbial profile: gram-negative organisms

	**Number of Patients**	**Microorganisms/Overall**
Gram-Negative Organisms	**16**	**34,04%**
Klebsiella	5	10,64%
Klebsiella oxytoca	4	8,51%
Klebsiella pneumoniae	1	2,13%
Pseudomonas aeruginosa	4	8,51%
Serratia marcescens	1	2,13%
Haemophilus influenzae	1	2,13%
Morexella	2	4,26%
Morexella Catarrhalis	1	2,13%
Morexella Nonliquefaciens	1	2,13%
Enterobakter cloacae	2	4,26%
Escherichia coli	1	2,13%

**Table 4:** Causative agents of infectious keratitis categorized by microbial profile: fungal organisms

	**Number of Patients**	**Microorganisms/Overall**
Fungi	**2**	**4,26%**
Candida	1	2,13%
Fusarium Sp.	1	2,13%

Empirical treatment was initiated in all patients based on clinical severity and suspected etiology. A total of 44 patients (60.2%) received fortified topical vancomycin (50 mg/mL) and ceftazidime (50 mg/mL), combined with bacitracin-neomycin ointment. Another 25 patients (34.2%) were treated with topical moxifloxacin hydrochloride (a fourth-generation fluoroquinolone) along with the same ointment.

Two patients with herpes simplex virus-associated keratitis (2.7%) received topical ganciclovir gel (1.5 mg/g) and moxifloxacin drops, both administered in combination with bacitracin-neomycin ointment. Two patients diagnosed with fungal keratitis (2.7%) were treated with fortified topical amphotericin B (0.5 mg/mL), moxifloxacin drops, and bacitracin-neomycin ointment, in addition to oral voriconazole (200 mg, twice daily). The bacitracin-neomycin ointment was uniformly included as adjunctive therapy in all treatment groups.

Of the 73 patients, 48 (65.8%) showed clinical resolution with topical medical therapy. Among these, 25 patients, most of whom had mild clinical presentations and negative culture results achieved improvement with a combination of topical moxifloxacin hydrochloride and bacitracin-neomycin ointment. An additional 20 patients responded to fortified vancomycin and ceftazidime combined with the same ointment. Two fungal keratitis cases were successfully managed with topical amphotericin B, moxifloxacin drops, and bacitracin-neomycin ointment. One patient with herpetic keratitis responded to topical ganciclovir gel, moxifloxacin, and bacitracin-neomycin ointment.

**Fig. 3:**
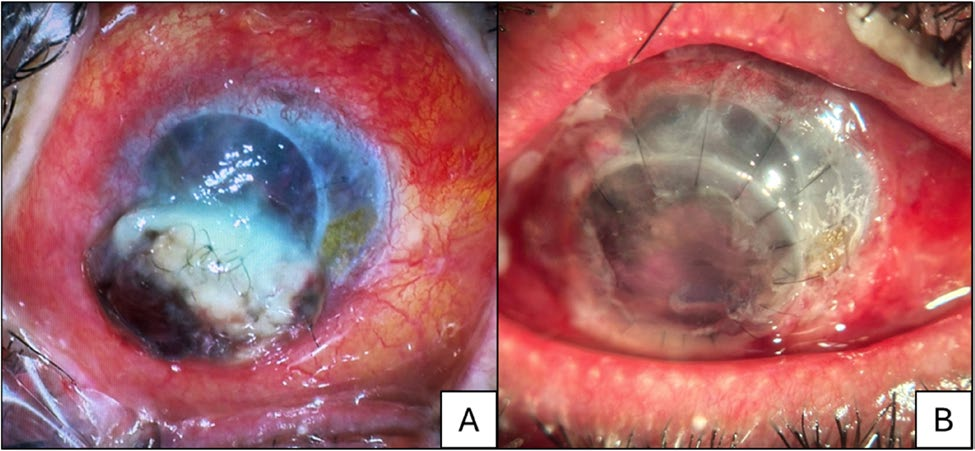
Clinical slit-lamp photographs of a post-keratoplasty patient with severe Gram-positive bacterial keratitis leading to graft failure and corneal perforation. **A** Preoperative presentation: Slit-lamp examination reveals extensive corneal melting with perforation at the inferior graft-host junction, secondary to microbial keratitis. The affected area demonstrates stromal necrosis, thinning, and anterior chamber collapse, indicative of an acute infective process with subsequent structural compromise. **B** Postoperative outcome following tectonic rPKP: Surgical intervention involved placement of an 8.25 mm donor graft inferonasally, with preservation of the superior clear graft segment. The adjacent conjunctival and scleral melt was concurrently managed via tectonic reconstruction to restore globe integrity. The image demonstrates proper graft position and stabilization of the previously necrotic zone.

PKP was performed in 13 patients (17.8%) whose clinical conditions deteriorated despite topical treatment. Additionally, 3 patients with prior keratoplasty underwent rPKP Fig. 3. Surgical intervention was declined by two patients.

Endophthalmitis developed in 7 patients (9.5%) as a complication of microbial keratitis. Among these, one case was polymicrobial, while the remaining six were monomicrobial. The identified causative organisms were *Staphylococcus spp.* in 44.4% of cases, *Streptococcus spp.* in 33.3%, *Klebsiella spp.* in 11.1%, and *Pseudomonas aeruginosa* in 11.1%.

A total of 7 patients (9.6%) required surgical intervention, including PPV or PPV combined with PKP supported by keratoprosthesis (KPro). Evisceration was performed in one of these cases. The anatomical success rate among surgically treated patients was 85.7%.

## Discussion

This study evaluated the clinical and microbiological characteristics of microbial keratitis cases treated at a tertiary eye care center in Türkiye. Previous reports have suggested that the average age of patients with monomicrobial and polymicrobial infections is generally similar [6, 7]. However, in the present study, the mean age was found to be significantly higher in the polymicrobial group compared to the monomicrobial group. A predominance of male patients was observed in both groups, which is consistent with previous epidemiological studies on microbial keratitis [7].

The study by Akova Budak et al. reported data on microbial keratitis cases requiring hospitalization [8]. According to the study, 10% of patients had a history of previous ocular surgery, 10% had a history of ocular trauma, and 5% had a history of contact lens use.

The microbial spectrum of keratitis is known to be influenced by multiple factors, including occupational exposure to corneal trauma, agricultural work, contact lens use, and socioeconomic conditions such as national income level [1]. In our study, trauma emerged as the most frequently observed risk factor, which may be related to the role of our hospital in the treatment of military personnel and war-injured patients. This finding is consistent with previous studies that have also identified trauma as the most common predisposing factor [9, 10].

Contact lens use was identified as the second most common risk factor, followed by a history of corticosteroid use, prior herpes simplex virus infection, and intensive care unit admissions associated with immunosuppression or exposure keratopathy. These findings are consistent with previously published literature [2].

Seven patients who had received only topical corticosteroid therapy were included in the study, as this was not expected to influence culture outcomes. The off-label use of topical corticosteroids without microbiological confirmation may have contributed to disease progression and increased the need for surgical intervention. Additionally, 24.7% of the patient cohort had diabetes mellitus, a systemic condition that may have led to delayed epithelial healing, prolonged recovery, and poorer visual outcomes in affected individuals. These findings underscore the importance of individualized risk assessment in patients with microbial keratitis.

The culture positivity rate in this study was 64.4%, which is relatively high compared to values previously reported in the literature [11]. Since cultures were only obtained from patients who met specific clinical criteria, the observed culture positivity rate may reflect a more severe subset of microbial keratitis cases rather than the full clinical spectrum. The most frequently isolated group of pathogens was Gram-positive bacteria, followed by Gram-negative bacteria and fungal organisms. This distribution is consistent with the findings of several previous studies [12–16].

Among all isolates, Staphylococcus epidermidis was the most commonly identified species (12.76%), which is consistent with previously published data [17]. Although Pseudomonas aeruginosa has been reported as the most common Gram-negative pathogen in microbial keratitis in many studies [13, 14, 16], Klebsiella spp. was the predominant Gram-negative isolate in the present study. This observation suggests that clinicians working in socioeconomically disadvantaged populations should maintain a higher index of suspicion for Klebsiella-associated keratitis. The Asia Cornea Society Infectious Keratitis Study reported that fungal pathogens are the predominant causative agents in developing countries, whereas bacterial pathogens are more commonly isolated in developed countries [10]. Similarly, a large-scale study conducted in southern China also demonstrated a predominance of fungal keratitis [18]. In contrast, fungal keratitis accounted for only 4.25% of cases in the present study, making it considerably less frequent than bacterial keratitis. The use of contact lenses is known to be a significant risk factor for the development of *Pseudomonas aeruginosa* keratitis [19]. But in the present study, P. aeruginosa was isolated in two neonates who had prolonged stays in the intensive care unit, suggesting that hospitalization-related factors may have contributed to the infection.

In line with previous studies, the findings of this study showed no significant difference in clinical efficacy between fourth-generation fluoroquinolone monotherapy and fortified antibiotic combination therapy in the treatment of bacterial keratitis [20-23]. Based on current observations, moxifloxacin resistance appears to be relatively low in our region, however, the potential for future resistance highlights the importance of continued antimicrobial surveillance.

Patients with antibiotic-resistant severe keratitis are at increased risk for complications such as corneal perforation, endophthalmitis, and panophthalmitis. In such serious cases, therapeutic PKP may be an effective intervention to achieve microbial eradication and preserve globe integrity [24]. In the present study, PKP was performed in 13 out of 25 patients with resistant and severe keratitis who did not respond to topical medical therapy. The anatomical success rates were comparable to those reported in the literature [25–27]. Repeat PKP was performed in three patients who had previously undergone PKP; some of these surgeries had been performed at the study center long ago, while others had been conducted at different medical institutions.

According to current literature, secondary endophthalmitis develops in approximately 4.4% of microbial keratitis cases with 27.6% of isolates being Gram-positive, 19.3% Gram-negative, and 7.2% fungal [28]. In our study, endophthalmitis developed in 7 patients (9.5%) due to microbial keratitis, of which one was polymicrobial and six were monomicrobial. The causative agents were Gram-positive in 77.8% and Gram-negative in 22.2% of the cases.

In a recent series evaluating outcomes of keratitis-associated endophthalmitis, anatomical success was achieved in 87.5% of cases treated with KPro-assisted PPV followed by PKP [29]. Similarly, in our study, seven patients underwent PPV or KPro-assisted PPV combined with PKP. Evisceration was required in one patient. The overall anatomical success rate observed in our cohort was consistent with previous reports, supporting the effectiveness of this surgical approach in severe or refractory cases.

The main limitation of this study is its retrospective design. Since patients with mild findings who were treated and recovered at external centers were not included in the culture sampling, the culture positivity rate may appear lower, while the pathogen virulence may seem higher than it actually is. Treatment approaches, the presence of systemic diseases such as diabetes, history of corticosteroid use, and the timing of surgical interventions were not standardized; this may have affected the outcomes. Finally, since the findings of this study are based solely on data from a single tertiary center, they may not be generalizable to other populations or regions.

In conclusion, Staphylococcus epidermidis was the most frequently isolated Gram-positive organism in our cohort, while Klebsiella oxytoca was identified as the predominant Gram-negative pathogen. Careful evaluation of patient-specific risk factors and selection of empiric therapy based on local pathogen profiles are important considerations in the management of microbial keratitis. Topical antibiotic therapy remains the cornerstone of initial treatment for bacterial keratitis. However, in cases unresponsive to medical therapy or at high risk of corneal perforation, early PKP performed prior to limbal involvement or full-thickness perforation is may be associated with better clinical outcomes in selected cases.

## Data Availability

No datasets were generated or analysed during the current study.
